# Advancing Colorectal Cancer Prevention in Inflammatory Bowel Disease (IBD): Challenges and Innovations in Endoscopic Surveillance

**DOI:** 10.3390/cancers17010060

**Published:** 2024-12-28

**Authors:** Ernesto Fasulo, Ferdinando D’Amico, Alessandra Zilli, Federica Furfaro, Clelia Cicerone, Tommaso Lorenzo Parigi, Laurent Peyrin-Biroulet, Silvio Danese, Mariangela Allocca

**Affiliations:** 1 Department of Gastroenterology and Endoscopy, IRCCS San Raffaele Scientific Institute, Vita-Salute San Raffaele University, 20132 Milan, Italy; fasulo.ernesto@hsr.it (E.F.); damico.ferdinando@hsr.it (F.D.); zilli.alessandra@hsr.it (A.Z.); furfaro.federica@hsr.it (F.F.); cicerone.clelia@hsr.it (C.C.); parigi.tommaso@hsr.it (T.L.P.); danese.silvio@hsr.it (S.D.); 2 Department of Gastroenterology, Nancy University Hospital, F-54500 Vandœuvre-lès-Nancy, France; l.peyrin-biroulet@chru-nancy.fr; 3 NSERM, NGERE, University of Lorraine, F-54000 Nancy, France; 4 INFINY Institute, Nancy University Hospital, F-54500 Vandœuvre-lès-Nancy, France; 5 FHU-CURE, Nancy University Hospital, F-54500 Vandœuvre-lès-Nancy, France; 6 Groupe Hospitalier Privé Ambroise Paré-Hartmann, Paris IBD Center, F-92200 Neuilly sur Seine, France; 7 Division of Gastroenterology and Hepatology, McGill University Health Centre, Montreal, QC H4A 3J1, Canada

**Keywords:** inflammatory bowel disease, IBD, ulcerative colitis, Crohn’s disease, colorectal cancer, CRC, screening, endoscopy, artificial intelligence

## Abstract

Patients with inflammatory bowel disease (IBD) face an elevated risk of developing colorectal cancer (CRC). Endoscopic surveillance is a cornerstone in CRC prevention, enabling early detection and intervention. However, despite recent advancements, challenges persist. Chromoendoscopy (CE), considered the gold standard for dysplasia detection, remains underutilized due to logistical constraints, prolonged procedural times, and the need for specialized training. New technologies, such as endomicroscopy, confocal laser endomicroscopy (CLE), and molecular endoscopy (ME), promise unprecedented precision in lesion characterization but are limited to specialized centers. Artificial intelligence (AI) can transform the field; however, barriers to widespread AI adoption include the need for robust datasets, real-time video integration, and seamless incorporation into existing workflows. Beyond technology, patient adherence to surveillance protocols, including bowel preparation and repeat procedures, remains a critical hurdle. This review aims to explore the advancements, ongoing challenges, and future prospects in CRC prevention for IBD patients, focusing on improving outcomes and expanding the implementation of advanced surveillance technologies.

## Simple Summary

Patients with inflammatory bowel disease (IBD), such as Crohn’s disease and ulcerative colitis, have a higher risk of developing colorectal cancer (CRC) due to chronic inflammation in the colon. Detecting early changes, such as dysplasia, through regular endoscopic surveillance is critical for preventing CRC in this high-risk population. This review explores the current strategies for CRC prevention in IBD, highlighting the use of endoscopic techniques like dye-based chromoendoscopy or virtual chromoendoscopy, and emerging technologies such as confocal laser endomicroscopy, molecular imaging, and artificial intelligence. While these innovations promise the improved detection and characterization of lesions, barriers such as limited access, operator training, and patient adherence to surveillance protocols still hinder their full potential. By addressing these challenges and optimizing surveillance programs, this paper aims to guide improvements in CRC prevention, ensuring more effective and accessible care for patients with IBD.

## 1. Introduction

Colorectal cancer (CRC) is the third most common cancer globally and a leading cause of cancer-related mortality, accounting for over 1.9 million new cases annually [1]. Among patients with inflammatory bowel disease (IBD), which includes ulcerative colitis (UC) and Crohn’s disease (CD), the risk of developing CRC is significantly elevated compared to the general population [2,3,4]. Chronic, persistent, and relapsing inflammation of the colonic mucosa drives the progression from inflammation to dysplasia and, ultimately, to malignancy [5,6]. The pathogenesis of IBD-associated CRC follows a distinct inflammation–dysplasia–carcinoma sequence, differing from the adenoma–carcinoma pathway observed in sporadic CRC [7,8]. Prolonged mucosal inflammation leads to oxidative DNA damage, epigenetic alterations, and genomic instability, which, over time, result in the clonal expansion of dysplastic cells and the development of malignancy [9,10]. Specific molecular pathways, such as alterations in tumor suppressor genes (e.g., TP53) and oncogenes (e.g., KRAS), are implicated in this process, alongside the disruption of epithelial barrier integrity and chronic immune activation [11,12]. Individuals with extensive colonic involvement face up to a two- to three-fold greater risk of CRC, with cumulative risks reaching approximately 2% after 10 years, 8% after 20 years, and up to 18% after 30 years of disease duration [13,14].

Endoscopic surveillance is the cornerstone of CRC prevention in IBD, offering the opportunity for early dysplasia detection and endoscopic or surgical intervention before malignancy develops [15,16]. However, the effectiveness of surveillance depends on several factors, including the quality of imaging, the ability to identify flat or subtle lesions, and the adherence to risk-based surveillance intervals [17]. Furthermore, the unique challenges posed by IBD-associated dysplasia (the multifocal nature or the difficulty of distinguishing dysplastic changes from inflammation) necessitate advanced techniques and highly trained endoscopists [18].

This review examines the current strategies for CRC prevention in IBD, focusing on endoscopic surveillance as a primary tool for risk mitigation. We explore the latest advancements in imaging technologies, the role of tailored surveillance protocols, and the impact of guidelines on clinical practice. By addressing the gaps and challenges in current approaches, we aim to provide a comprehensive framework for optimizing CRC prevention in this high-risk population.

## 2. Standard Endoscopic Practice

**Figure 1:**
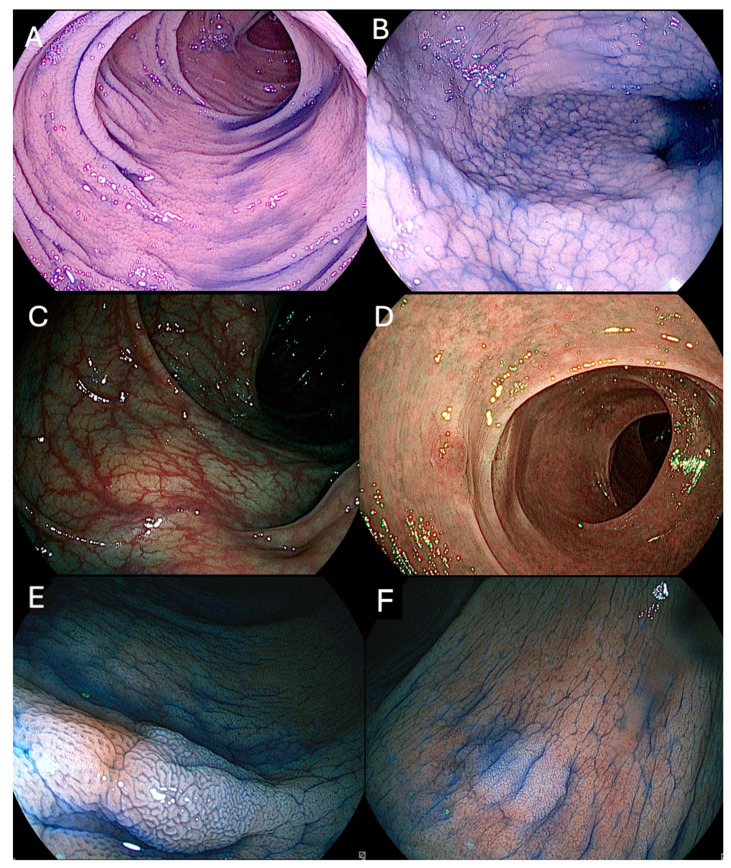
Endoscopic images during surveillance for inflammatory bowel diseases (IBD). (**A**,**B**) Dye-based chromoendoscopy (DBE). (**C**,**D**) Virtual chromoendoscopy (VCE). (**E**,**F**) Combination of both DCE and VCE to enhance visualization of a non-polypoid lesion. The copyright of the image belongs to the authors.

Over the years, substantial advancements in endoscopic technologies have improved the ability to identify and characterize dysplasia in IBD population. Both white-light endoscopy (WLE) and chromoendoscopy (CE) remain cornerstone techniques, each with strengths and limitations (Figure 1).

### 2.1. White Light Endoscopy

WLE has historically been the standard method for surveillance in IBD [19,20]. In its earlier form, standard-definition WLE (SD-WLE) relied on four-quadrant random biopsies taken every 10 cm along the colon to detect dysplasia. This approach was labour-intensive and limited, as it sampled only about 1% of the colonic mucosa, often missing flat or subtle lesions [21]. The advent of high-definition WLE (HD-WLE) has dramatically improved surveillance by offering sharper images, better resolution, and enhanced mucosal visualization [22]. These advancements allow for the more reliable detection of visible dysplastic lesions. Restricted evidence suggests that HD-WLE might be comparable to dye-based CE in certain settings. For instance, a randomized clinical trial (RCT) reported no significant difference in dysplasia detection between HD-WLE and DCE, with detection rates of 18.9% and 17.8%, respectively (*p =* 0.91) [23]. Furthermore, HD-WLE has reduced the reliance on random biopsies by enabling targeted biopsies, which have shown similar efficacy in detecting dysplasia [24]. This shift minimizes the procedural burden while maintaining diagnostic accuracy. However, HD-WLE is not without limitations. Flat or non-polypoid lesions, particularly in areas of significant inflammation or scarred mucosa, may still be missed [25]. Consequently, HD-WLE is often complemented by advanced imaging techniques such as dye-based chromoendoscopy (DCE) or virtual chromoendoscopy (VCE) to enhance lesion detection and improve diagnostic yield [26].

### 2.2. Chromoendoscopy

CE, particularly DCE, is widely regarded as the gold standard for dysplasia detection in IBD [27,28]. This technique involves the application of contrast-enhancing dyes, such as methylene blue or indigo carmine, to the colonic mucosa. These dyes highlight subtle mucosal changes, such as architectural distortions or vascular abnormalities, that may be missed by WLE, allowing for the more precise detection of dysplastic lesions [29]. Numerous studies have highlighted the superiority of DCE over SD-WLE. A meta-analysis demonstrated that DCE increased the diagnostic yield for dysplasia by 7% (95% CI: 3.2–11.3) compared to SD-WLE [6]. Moreover, a prospective study found that DCE identified significantly more dysplastic lesions than HD-WLE, detecting 17 lesions compared to 7 in 305 patients (*p =* 0.032) [30]. Similarly, a multicenter trial reported that DCE provided a 57.4% incremental yield for dysplasia detection compared to WLE, even when performed sequentially during the same procedure (*p* < 0.001) [31]. An updated meta-analysis of 6 RCTs further confirmed that DCE detects significantly more dysplasia than HD-WLE (18.8% vs. 9.4%; *p =* 0.006), with an OR of 1.94 (95% CI: 1.21–3.11), supporting its role as a superior strategy in high-risk patients [32]. Despite its proven efficacy, the widespread adoption of DCE in clinical practice remains limited. This is largely due to its longer procedural time, dependence on operator expertise, and the need for specialized training. A survey among gastroenterologists revealed that 26.5% viewed DCE as the preferred method for dysplasia detection, with barriers including a prolonged procedure duration (46.9%) and insufficient training (40.8%) [33]. VCE has emerged as a promising alternative to DCE, utilizing digital image enhancement technologies to mimic the effects of dye-based endoscopy without the need for dye application [34]. Techniques such as narrow-band imaging (NBI), iSCAN, blue laser imaging/linked colour imaging (BLI/LCI), and flexible spectral imaging colour enhancement (FICE) enhance mucosal visualization by digitally altering image contrast and highlighting vascular and mucosal patterns in real time [35]. These technologies reduce hte procedural complexity and time, offering an efficient alternative to DCE [36]. For instance, a meta-analysis found no significant difference in dysplasia detection rates between VCE and DCE, but VCE required an average of 7 min less procedural time per examination (*p* < 0.001) [37]. However, data comparing VCE to DCE remain mixed. While VCE offers advantages in terms of efficiency and ease of use, its diagnostic accuracy may vary depending on the specific technology employed and the expertise of the operator. Further studies are needed to determine whether VCE can fully replace DCE in all clinical scenarios.

### 2.3. International Guidenlines

Surveillance should begin 8 years after symptom onset in patients with UC with an extension beyond the rectum or CD with colonic involvement of at least one-third of the organ. Surveillance intervals are tailored to individual risk factors. High-risk patients, such as those with active inflammation, prior dysplasia, or a family history of CRC, should undergo colonoscopies every 1–3 years, while low-risk patients may extend the intervals to up to 5 years [38]. In recent years, the role of random biopsies has decreased substantially [39]. While previously a cornerstone of surveillance, random biopsies are now discouraged in average-risk patients undergoing CE or HD-WLE with targeted biopsies, as their diagnostic yield is low [40]. Current international guidelines reflect some heterogeneity in their recommendations for surveillance strategies. The Surveillance for Colorectal Endoscopic Neoplasia Detection and Management in Inflammatory Bowel Disease Patients: International Consensus (SCENIC) prioritizes HD-WLE and CE for all patients and emphasizes targeted biopsies, reserving random biopsies for high-risk groups like PSC patients or those with prior dysplasia or an atrophic colon [28,41]. The American Gastroenterological Association (AGA) guidelines suggest DCE as the preferred method but accept VCE as an alternative [27]. Random biopsies are recommended only for high-risk patients or when CE is unavailable. Similarly, the European Crohn’s and Colitis Organisation (ECCO) guidelines prioritize HD-WLE, DCE, or VCE with targeted biopsies as the primary approach and advise random biopsies only in select high-risk cases [42]. In contrast, the British Society of Gastroenterology (BSG) guidelines advocate for HD-WLE as the standard method for surveillance [43]. When only SD-WLE is available, DCE is strongly recommended to enhance dysplasia detection, while VCE is not advised as a first-line method.

## 3. Optical Diagnosis Training for IBD

There are no specific additional prerequisites required to begin training in optical diagnosis for IBD-related neoplasia, apart from general endoscopic skills [44] (Table 1). However, the interpretation of CE findings in IBD poses unique challenges due to the inflammatory background, which can make dysplasia difficult to identify and lead to prolonged procedure times or unnecessary biopsies. To address this, the European Society of Gastrointestinal Endoscopy (ESGE) recommends that all endoscopists performing surveillance in IBD patients undergo dedicated training in optical diagnosis, ideally under the supervision of an expert in IBD-related lesion detection [44,45,46,47]. Training should include a combination of hands-on experience, instructional materials such as atlases and videos, and interactive web-based learning platforms [44,48]. Training begins with mastering DCE, where endoscopists gain experience in identifying lesions and performing targeted biopsies under expert supervision. During this phase, random biopsies serve as a safety measure to ensure thorough surveillance while the endoscopist builds confidence and accuracy in lesion identification. Over time, as proficiency is established and validated through histological feedback, reliance on random biopsies can be reduced in favour of targeted sampling alone. Once competence in DCE is achieved, endoscopists can transition to VCE.

### 3.1. Transitioning to VCE

As said before, VCE represents an alternative to DCE. Studies suggest VCE, when used with technologies such as NBI or LCI, can achieve a comparable efficacy in detecting dysplasia [37,49]. A gradual transition to VCE is recommended, ensuring that endoscopists are well-trained and confident in its use before fully adopting it as their primary surveillance method [44].

### 3.2. Competence and Maintenance

Competence in optical diagnosis is achieved when endoscopists can reliably detect neoplasia in IBD patients, supported by histological confirmation. ESGE suggests that a benchmark for competence is a neoplasia detection rate of at least 10% in IBD surveillance cases, reflecting real-world detection rates in high-risk populations [31,44]. To maintain proficiency, endoscopists should regularly review and audit their performance, including an annual review of at least 10 IBD-related lesions. For those who do not perform IBD surveillance regularly, refresher training may be necessary to sustain their skills.

### 3.3. Classification Systems for Dysplasia Detection

Although no fully validated classification system exists for IBD-related neoplasia, frameworks such as Kudo’s pit pattern classification and the Frankfurt Advanced Chromoendoscopic IBD Lesions (FACILE) classification provide useful guidance [50,51]. Kudo’s classification, while originally developed for colorectal polyps, offers a high negative predictive value (NPV) (88–94%) for non-dysplastic lesions in IBD [31,52]. The FACILE classification, which incorporates features such as flat morphology, irregular surfaces, and abnormal vasculature, has shown promise in identifying dysplasia, with an area under the curve (AUC) of 0.76 [51]. These tools, combined with high-definition imaging, assist endoscopists in refining their diagnostic accuracy during surveillance.

## 4. Advanced Endoscopic Imaging Technologies

**Figure 2:**
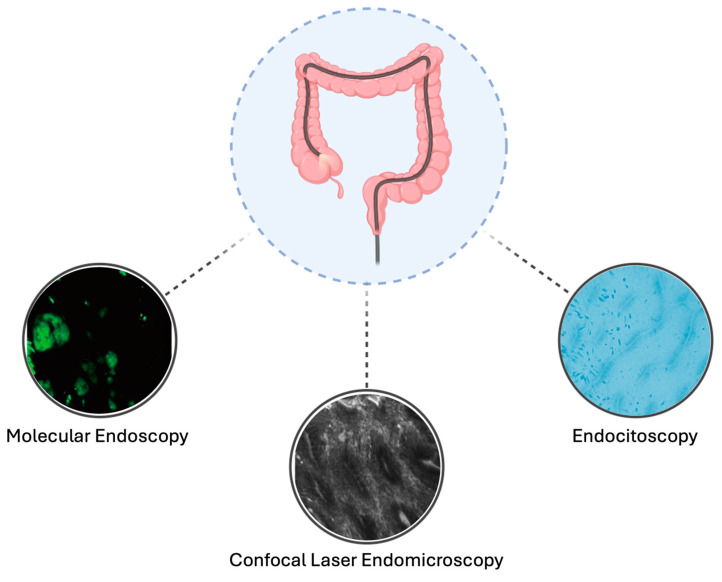
Overview of advanced endoscopic imaging technologies used for dysplasia detection in patients with inflammatory bowel disease (IBD). These technologies include molecular endoscopy (ME), confocal laser endomicroscopy (CLE), and endocytoscopy. Each modality offers unique advantages for identifying dysplastic lesions in IBD patients, improving diagnostic accuracy.

Advanced endoscopic imaging technologies, including endocytoscopy, confocal laser endomicroscopy (CLE), and molecular endoscopy (ME), offer innovative solutions by enhancing lesion detection, improving visualization, and providing real-time insights into tissue architecture and molecular alterations [53] (Table 2, Figure 2).

### 4.1. Endocytoscopy

Endocytoscopy is an ultra-high magnification endoscopic technique capable of visualizing cellular and subcellular structures directly in vivo [54]. By achieving a magnification of up to 1400×, it allows for the detailed assessment of crypt architecture, cellular nuclei, goblet cells, and vascular patterns, effectively functioning as a “virtual histology” tool. To optimize visualization, mucolytic agents such as N-acetylcysteine are often applied to clear the mucus layer, followed by topical dyes like methylene blue or toluidine blue to highlight cellular structures. Although the use of endocytoscopy in clinical practice remains sporadic, emerging case reports demonstrate its potential utility in IBD surveillance. For instance, a case study by Fukunaga et al. described how endocytoscopy was employed to visualize cellular-level abnormalities. This approach successfully identified atypical crypt architecture suggestive of dysplasia, enabling targeted biopsy and subsequent histopathological confirmation [55]. Similarly, Misawa et al. reported two cases of colitis-associated neoplasia where endocytoscopy provided a detailed visualization of neoplastic changes, including irregular crypt patterns and nuclear abnormalities, not easily discernible with standard endoscopy [56]. These findings were pivotal for diagnosing early-stage neoplasia and guiding therapeutic decisions. Furthermore, a pilot study by Kudo et al., which analyzed 103 lesions from 62 patients (23 UC-associated neoplasia and 80 non-neoplastic lesions), demonstrated that combining the pit pattern analysis with an endocytoscope-based assessment of irregularly shaped nuclei (the EC-IN-PIT strategy) achieved greater accuracy in predicting UC-associated neoplasia compared to using pit pattern analysis alone (88% vs. 67%, *p* < 0.01) [57].

### 4.2. Confocal Laser Endomicroscopy

CLE is a powerful optical imaging technique that combines laser scanning technology with the use of fluorescent dyes (e.g., intravenous fluorescein) to provide high-resolution, real-time images of the mucosa at a depth of up to 250 μm [58]. CLE allows for the detailed visualization of crypt architecture, vascular patterns, and epithelial structures, enabling clinicians to identify dysplastic changes and neoplastic transformations with high accuracy [59,60]. For instance, Kiesslich et al. showed that CLE, when combined with chromoendoscopy, significantly improved the diagnostic yield for dysplasia compared to random biopsies alone, with a sensitivity of 94.7% and specificity of 98.3%, using histology as the reference standard [61]. Similarly, a study by Rispo et al. reported 86% sensitivity and 100% specificity for CLE in identifying dysplasia in long-standing UC, underscoring its potential to enhance surveillance protocols [62]. These findings highlight CLE’s ability to pinpoint lesions that may otherwise remain undetected, especially those with flat or subtle features. Studies have demonstrated that CLE, by targeting biopsies to suspicious areas identified during the procedure, can achieve comparable or superior detection rates while significantly reducing the number of biopsies required by fivefold without compromising diagnostic accuracy [61]. Furthermore, specific comparisons between CLE and standard techniques in IBD populations are limited. Dlugosz et al. demonstrated that CLE-targeted biopsies detected additional endoscopically invisible dysplasia compared to HD-WLE alone, with CLE achieving 89% sensitivity, 96% specificity, and a 99% NPV for dysplasia detection [63]. When compared, CE and CLE both demonstrated 100% sensitivity and NPV for intraepithelial neoplasia (IEN), with CLE showing a slightly higher specificity (98.4% vs. 96.8%) and positive predictive value (PPV) (66.7% vs. 62.5%) [64]. A multicenter study on CD surveillance reported that combining CE with CLE achieved 86.7% accuracy, 42.9% sensitivity, and 92.4% specificity for dysplasia detection, compared to 80.3%, 28.6%, and 86.4% for CE alone. However, the study highlighted frequent equipment failures, limiting CLE’s practicality in routine practice [65]. Despite its potential advantages, the use of CLE in IBD surveillance is not without limitations. While it excels at identifying dysplasia in flat mucosa, its effectiveness can be hindered by technical challenges, such as difficulty stabilizing the scope for imaging protruding polyps, and its dependence on operator expertise. Additionally, the integration of CLE into routine practice is limited by the high cost of the equipment and the need for extensive training to ensure the accurate interpretation of CLE findings [66]. Interobserver variability remains a concern, with reported kappa values for agreement on dysplasia detection ranging between 0.47–0.94, depending on the study and level of operator experience [67,68].

### 4.3. Molecular Endoscopy

ME represents the next frontier in dysplasia surveillance, combining advanced imaging techniques with targeted molecular probes to visualize specific biomarkers in the gastrointestinal mucosa [69]. This approach leverages fluorescently labelled antibodies or peptides that bind to dysplasia-related proteins, enabling the real-time detection of molecular alterations associated with neoplastic transformation [70]. Gounaris et al. demonstrated that cathepsin-based fluorescent probes could distinguish dysplastic lesions from inflamed tissue in colitis-induced murine models, achieving significantly brighter near-infrared fluorescence signals in dysplastic areas (*p* < 0.001) [71]. Similarly, Mitsunaga et al. showed that γ-glutamyl hydroxymethyl rhodamine green (gGlu-HMRG), a cancer-specific fluorescence probe, allowed the enhanced visualization of colitis-associated cancers, providing a high cancer-to-background contrast in murine models (*p* < 0.01) [72]. De Palma et al. further demonstrated the feasibility of ex vivo fluorescence staining using a peptide-based molecular probe, combined with CLE, to predict dysplasia in UC. This approach improved lesion characterization and guided resectability decisions (*p* < 0.05) [73]. While this technique shows great potential, its application remains limited to expert centers due to high costs and training requirements. ME, however, holds promise for advancing precision surveillance in IBD [74,75].

## 5. Artificial Intelligence in Endoscopic Surveillance

The integration of artificial intelligence (AI) into endoscopic surveillance for IBD represents a promising advancement to enhance the detection and characterization of dysplastic and neoplastic lesions in this complex scenario [76,77]. The unique challenges of surveillance in IBD patients, such as the presence of background inflammation and the often flat and subtle nature of associated dysplastic lesions, necessitate advanced diagnostic tools to optimize monitoring strategies [76,78].

Recent studies have demonstrated the potential of AI algorithms, particularly convolutional neural networks (CNNs), to accurately differentiate neoplastic from non-neoplastic lesions in IBD [79,80].

In a study by Yamamoto et al., a CNN-based AI system was trained on non-magnified endoscopic images to classify IBD-associated neoplasia (IBDN) into two groups: adenocarcinoma/high-grade dysplasia (HGD) and low-grade dysplasia/sporadic adenoma/normal mucosa [79]. Using a dataset of 862 images expanded to 6.3 million through augmentation, the AI achieved a sensitivity of 72.5%, specificity of 82.9%, and accuracy of 79.0%**.** These results surpassed the sensitivity of 60.5% and accuracy of 77.8% of expert endoscopists, as well as the performance of non-experts, who achieved a sensitivity of 70.5%, specificity of 78.8%, and accuracy of 75.8%. This highlights the potential of AI to support clinical decision-making by offering higher sensitivity and accuracy than experts and non-experts. However, this study focused on non-magnified images, limiting its generalizability to real-world endoscopic workflows. Another promising AI model for detecting colorectal lesions in IBD was developed and retrained using HD-WLE and CE images [80]. Initially trained on datasets of non-IBD-associated polyps, the algorithm was retrained with 1266 HD-WLE and 426 DCE images of IBD-associated lesions. The retrained model showed exceptional performance for HD-WLE images, with a sensitivity of 95.1%, specificity of 98.8%, PPV of 98.9%, NPV of 94.7%, accuracy of 96.8%, and an area under the curve (AUC) of 0.85. This performance significantly improved upon the original model’s sensitivity of 50% and accuracy of 64%. The model also demonstrated promising results when applied to CE images, although its overall performance was lower, with a sensitivity of 67.4%, specificity of 88.0%, PPV of 83.3%, NPV of 74.3%, accuracy of 77.8%, and an AUC of 0.65. Subgroup analysis revealed that IBD-Computer Aided Detection (CADe) achieved high sensitivity across lesion sizes, detecting lesions ≤ 5 mm with 93% sensitivity, lesions 6–10 mm with 91% sensitivity, and those >10 mm with 85% sensitivity. Notably, most lesions missed were in areas of minimal inflammation (Mayo endoscopic subscore 0–1), suggesting that the AI’s performance could vary depending on the inflammatory environment

These findings highlight the potential of AI to address specific challenges in IBD surveillance; however, further studies are needed to validate the effectiveness of AI systems in clinical practice and to overcome current limitations.

## 6. Optimization of Endoscopic Surveillance Protocols

### 6.1. Sedation

Individuals with IBD typically require a greater number of colonoscopies over their lifetimes. Among this population, sedation and pain management are particularly important, as studies suggest that IBD patients experience more procedural discomfort and report lower satisfaction with sedation compared to those undergoing colonoscopy for CCR screening [81,82]. Patients with CD and UC also tend to view endoscopy as less acceptable than other methods used for disease monitoring and diagnosis [83]. Additionally, IBD has been identified as a condition associated with higher demands for sedation and analgesia during the endoscopic procedure [84,85]. According to Weber et al., on average, colonoscopies in IBD patients last significantly longer than those in non-IBD patients (22.7 vs. 17.2 min, *p* < 0.01), often due to strictures, inflammation, and altered anatomy due to prior surgeries, which affect procedural ease and patient comfort [86]. Sedation requirements in IBD patients are notably higher. For procedures using Intravenous Conscious Sedation (IVCS), IBD patients required significantly larger doses of midazolam (5.7 vs. 4.3 mg, *p* < 0.01) and opioids (157.6 vs. 119.4 µg fentanyl equivalents, *p* < 0.01) compared to non-IBD patients [86]. The demand for Monitored Anesthesia Care (MAC) is also significantly higher in IBD patients, utilized in 73.1% of cases compared to just 13.2% in non-IBD patients (*p* < 0.01). Moreover, it is important to note that IBD patients, particularly those with long-standing inflammation, face significantly higher cardiovascular risks compared to the general population [87,88]. Severe inflammation, as observed in IBD, has been linked to endothelial dysfunction, increased arterial stiffness, and prothrombotic states, further compounding the challenges associated with sedation and procedural management [89,90]. Given the higher sedation and analgesia requirements for individuals with IBD, achieving effective and safe sedation during colonoscopy is essential. Propofol is a highly valuable agent in this context due to its rapid onset, predictable effects, and ability to provide deep sedation with a favourable safety profile [91,92]. Importantly, recent evidence suggests that propofol can be safely administered by trained gastroenterologists, expanding its accessibility and utility in routine endoscopic practice [93,94,95]. A recent study demonstrated the safety and tolerability of non-anesthesiologist-administered propofol (NAAP) using target-controlled infusion in a large cohort of 18,302 gastrointestinal endoscopic procedures. Among these, 11,140 were colonoscopies, with adverse events reported in only 1.3% of cases [96]. Moreover, 98.9% of patients expressed willingness to repeat the procedure with the same sedation protocol, underscoring high patient satisfaction. The use of propofol with NAAP has also been specifically studied in IBD patients. In an RCT comparing propofol deep sedation with NAAP to moderate sedation with midazolam and fentanyl in individuals with IBD, NAAP significantly improved satisfaction scores (mean 60.1 vs. 51.2; *p* < 0.001) assessed via a 13-item Likert-scale questionnaire [97]. Patients receiving NAPS reported less pain, greater amnesia, fewer procedural disruptions, and a strong preference for the same sedation in future colonoscopies. No safety concerns were identified, highlighting propofol’s efficacy and tolerability.

### 6.2. Bowel Preparation

Adequate bowel preparation is a critical prerequisite for successful colonoscopy in IBD patients as it ensures an accurate disease assessment and reliable dysplasia or cancer surveillance. However, bowel preparation in this group of individuals presents unique challenges due to the nature of the diseases, including inflammation, strictures, altered anatomy from prior surgeries, and risk of mucosal damage, especially in cases of active disease.

Several factors have been identified as predictors of poor bowel preparation in IBD patients. Studies have shown that male sex, non-split dosing regimens, older age, and poor patient compliance significantly increase the risk of suboptimal preparation [98]. For example, split-dosing regimens have consistently been associated with a better preparation quality, with Maida et al. reporting an 85.4% success rate using a 1 L polyethylene glycol (PEG-)aspartate split regimen compared to lower success rates in non-split protocols (*p* < 0.05) [98]. Kumar et al. also highlighted that moderate-to-severe disease activity and biological therapy were independent predictors of poor preparation in IBD patients, while age >65 years further increased the risk [99]. Additionally, patients with active CD reported more abdominal pain during preparation, which may further reduce adherence [100].

Current evidence on bowel preparation regimens tailored specifically for IBD patients, particularly those with severe phenotypes such as perianal disease or stenosing CD, is limited. A systematic review and meta-analysis found a paucity of data comparing different regimens, particularly in high-risk subgroups [101]. While high- and low-volume PEG-based solutions showed a similar efficacy for adequate bowel cleansing, patient tolerability was higher for low-volume PEG regimens supplemented with simethicone or adjuvants like ascorbate or bisacodyl. For example, Kim et al. reported a 92.9% success rate with a 2 L PEG-aspartate solution compared to 96.2% with a 4 L PEG-electrolyte lavage solution (ELS) solution, but patients in the low-volume group experienced significantly fewer side effects such as nausea (*p* < 0.05) [102]. These findings highlight the need for further head-to-head studies comparing these regimens in specific IBD subgroups to optimize patient outcomes.

## 7. Discussion

Technological advancements in endoscopy have brought significant improvements to the surveillance of IBD patients. Scopes with enhanced resolution, wider visual fields, and greater magnification capabilities have revolutionized the ability to detect and characterize dysplastic lesions [103]. Despite these technological advancements, clinical practice often falls short of guideline recommendations. CE, for instance, while highly effective, remains underutilized in routine clinical settings, with limited adoption outside referral centers. Several barriers also contribute to this gap, including the need for specialized training, longer procedural times, and logistical challenges. Moreover, while studies on CE primarily focus on dysplasia detection, quantifying its impact on reducing CRC incidence and related mortality remains challenging, making it difficult to assess these long-term outcomes. Emerging technologies, such as CLE, endocytoscopy, and ME, offer promising opportunities to improve the accuracy of dysplasia and early cancer detection while reducing the reliance on random biopsies. These tools enable real-time “virtual histology”, providing a detailed assessment of cellular and subcellular structures. However, their use remains largely confined to research settings and centers of excellence due to high costs, technical complexity, and the need for advanced operator training. Importantly, there is a lack of robust real-world data supporting their feasibility outside academic centers, and comparative studies against CE—the current gold standard—are scarce. Furthermore, cost-effectiveness analyses are insufficient, leaving critical questions about their economic viability unanswered. Without rigorous evidence demonstrating their impact on clinical outcomes and CRC prevention, these technologies remain far from routine clinical application, highlighting the need for further research to assess their practicality and benefits in everyday practice. AI represents another promising frontier in endoscopic surveillance, having already shown significant potential in CRC detection within the general screening population, and it is quite well integrated into clinical practice [104,105,106]. However, its application in IBD surveillance remains in its infancy, with few models specifically designed to address the unique challenges of this population. AI has the potential to enhance diagnostic accuracy, reduce inter-operator variability, and optimize surveillance protocols, but its implementation requires robust validation in real-world clinical settings. One major hurdle in the implementation of AI in IBD surveillance is the ability to analyse full-motion videos and distinguish informative frames from non-informative ones in real time [107]. Developing AI systems capable of processing live video feeds and providing instant feedback to endoscopists is crucial for its practical application in IBD surveillance. Another challenge lies in the limited availability of large, diverse, and well-annotated datasets specific to IBD-associated dysplasia. Moreover, the generalizability of AI systems across different patient populations, endoscopic equipment, and imaging modalities needs to be established, as they are often trained on data from a single center or a specific endoscopic system. Furthermore, the integration of AI into the existing clinical workflows poses logistical and regulatory challenges. The deployment of AI systems in real-world practice requires seamless integration with endoscopic equipment and electronic health record systems. Additionally, the medicolegal implications of AI-assisted decision-making, such as liability in case of missed or misclassified lesions, must be carefully considered and addressed. While technological advancements are essential, the success of surveillance programs also hinges on patient adherence. A lack of understanding regarding the importance of surveillance and the discomfort associated with procedures can significantly reduce participation rates. Sedation plays a crucial role in improving the patient experience, particularly for IBD patients who often undergo multiple colonoscopies over their life. Propofol represents an excellent option for sedation in IBD surveillance. However, its administration by gastroenterologists is not universally recognized or permitted under current legislative frameworks, which can limit its availability in certain settings. The introduction of novel sedatives, such as Remimazolam, which offers similar advantages with an even better safety profile and reduced risk of adverse events, could further enhance procedure acceptability and improve adherence, particularly in centers where NAAP is restricted [108,109,110].

Bowel preparation remains a critical yet underappreciated challenge in IBD surveillance. Standard regimens often fail to address the unique needs of IBD patients, particularly those with active inflammation, strictures, or post-surgical anatomical changes. For instance, stenosing CD or IPAA require tailored approaches. The use of a low-volume PEG, in combination with adjunctive agents like simethicone or prokinetics, to improve mucosal visualization and tolerability should be considered in a surveillance setting. Finally, a standardized risk stratification system for IBD patients is needed to tailor surveillance programs to individual risk profiles. Stratifying patients into high-, intermediate-, and low-risk categories would allow for more targeted resource allocation, ensuring intensive monitoring for high-risk individuals while reducing unnecessary interventions for those at lower risk. This approach would optimize resource utilization, improve clinical outcomes, and reduce the psychological and physical burden on patients.

## 8. Conclusions

Advancements in CRC surveillance for IBD patients have improved dysplasia detection. While CE remains the gold standard, advanced techniques like CLE, endocytoscopy, and ME offer opportunities for high-level patient care but are limited by cost and accessibility. Challenges such as inadequate bowel preparation, optimal sedation, and patient adherence continue to impact outcomes. Addressing these barriers through tailored preparation and sedation protocols, alongside personalized surveillance strategies, is crucial to optimizing CRC prevention in this high-risk population.

## Figures and Tables

**Table 1:** Optical diagnosis training for inflammatory bowel disease (IBD) steps according to the European Society of Gastrointestinal Endoscopy (ESGE) [44].

Aspect	Requirement/Details
Pre-Adoption Requirements	- Competence in HD colonoscopy using DCE or VCE with targeted biopsies.
Training Steps	- Onsite Training: 1-week course with an IBD optical diagnosis expert.
	- Self-Learning: Perform at least 20 pan-chromoendoscopy procedures with 20 targeted biopsies reviewed via histological feedback.
	- Incorporate random biopsies (every 10 cm) during the learning phase to ensure accuracy.
Competence Assessment	- Achieve a neoplasia detection rate of ≥10% in at least 20 IBD pan-chromoendoscopy procedures with targeted biopsies only.
Maintaining Competence	- Perform an in vivo audit of ≥10 IBD lesions annually.
	- If insufficient cases are performed, repeat training and learning phases.
Transition to VCE	- Progress gradually from DCE to VCE.
	- Utilize VCE after achieving competence in DCE.

IBD = Inflammatory Bowel Disease; HD = High Definition; DCE = Dye-based Chromoendoscopy; VCE = Virtual Chromoendoscopy.

**Table 2:** Comparison of advanced endoscopic imaging technologies for dysplasia detection in inflammatory bowel disease (IBD).

Technology	Key Features	Advantages	Limitations	Clinical Applications
Endocytoscopy	Ultra-high magnification (up to 1400×)	Provides “virtual histology” by visualizing cellular and subcellular structures in vivo	Requires mucolytic agents and topical dyes Limited availability High operator dependency	Early detection Targeted biopsies
Confocal Laser Endomicroscopy	High-resolution imaging up to a depth of 250 μm Utilizes fluorescent dyes	High sensitivity and specificity for dysplasia detection Enables real-time characterization	Highly expensive Extensive training Interobserver variability	Early detection Targeted biopsies Enhanced detection of flat lesions
Molecular Endoscopy	Combines imaging with molecular probes	Visualizes specific biomarkers Real-time detection of molecular alterations	Highly expensive Limited to expert centers Extensive training	Precision surveillance of dysplasia Research and experimental applications
